# Human‐wildlife conflict at high altitude: A case from Gaurishankar conservation area, Nepal

**DOI:** 10.1002/ece3.11685

**Published:** 2024-07-08

**Authors:** Abhinaya Pathak, Saneer Lamichhane, Maheshwar Dhakal, Ajay Karki, Bed Kumar Dhakal, Madhu Chetri, Jeffrey Mintz, Prakash Pun, Pramila Neupane, Tulasi Prasad Dahal, Trishna Rayamajhi, Prashamsa Paudel, Ashim Thapa, Pramod Raj Regmi, Shankar Thami, Ganesh Thapa, Suraj Khanal, Supriya Lama, Jenisha Karki, Sujan Khanal, Arockia E J Ferdin

**Affiliations:** ^1^ Department of National Parks and Wildlife Conservation Babarmahal, Kathmandu Nepal; ^2^ Department of Ecology, Behavior and Evolution, School of Biological Sciences University of California San Diego California USA; ^3^ Department of Wildlife Ecology and Conservation, School of Natural Resources and Environment University of Florida Gainesville Florida USA; ^4^ Department of Zoology and Physiology, Haub School of Environment and Natural Resources University of Wyoming Laramie Wyoming USA; ^5^ Gaurishankar Conservation Area Project, Head Office Singati, Dolakha Nepal; ^6^ Department of Natural Resources and the Environment Cornell University Ithaca New York USA; ^7^ Central Department of Zoology Tribhuwan University Kritipur Nepal; ^8^ Division Forest Office Charikot, Dolakha Nepal; ^9^ Kathmandu Forestry College Koteshwor, Kathmandu Nepal; ^10^ Institute of Forestry, Pokhara Campus, Hariyokharka Pokhara Nepal; ^11^ Department of Natural Resources and Environmental Studies, College of Environmental Studies and Oceanography National Dong Hwa University Hualien Taiwan

**Keywords:** coexistence, compensation policy, Gaurishankar conservation area, high altitude, Himalayan black bear, human‐wildlife conflict, leopard

## Abstract

Human–wildlife conflict studies of high‐altitude areas are rare due to budget constraints and the challenging nature of research in these remote environments. This study investigates the prevalence and increasing trend of human–wildlife conflict (HWC) in the mountainous Gaurishankar Conservation Area (GCA) of Nepal, with a specific focus on leopard (*Panthera pardus*) and Himalayan black bear (*Ursus thibetanus laniger*). The study analyzes a decade of HWC reports and identifies goats as the livestock most targeted by leopards. The Dolakha district of GCA received the highest number of reports, highlighting the need for mitigation measures in the area. In GCA, livestock attacks accounted for 85% of compensation, with the remaining 15% for human injuries. We estimate that the number of reported wildlife attacks grew on average by 33% per year, with an additional increase of 57 reports per year following the implementation of a new compensation policy during BS 2076 (2019 AD). While bear attacks showed no significant change post‐rule alteration, leopard attack reports surged from 1 to 60 annually, indicating improved compensation may have resulted in increased leopard‐attack reporting rates. The findings emphasize the economic impact of HWC on local communities and suggest strategies such as increasing prey populations, promoting community education and awareness, enhancing alternative livelihood options, developing community‐based insurance programs, and implementing secure enclosures (corrals) to minimize conflicts and foster harmonious coexistence. This research addresses a knowledge gap in HWC in high‐altitude conservation areas like the GCA, providing valuable insights for conservation stakeholders and contributing to biodiversity conservation and the well‐being of humans and wildlife.

## Graphical abstract

Recent policy changes have reduced hurdles to receiving compensation for wildlife attacks on people and livestock within the mountainous Gaurishankar Conservation Area of the Himalayas. We show that over the last decade, the number of reported attacks have increased by approximately 33% per year, after accounting for the boost in reporting due to improved compensation. The reported number of injuries by Himalayan black bears was overshadowed by a rise in reports of leopard predation on livestock, particularly goats, an important source of income for local farmers.

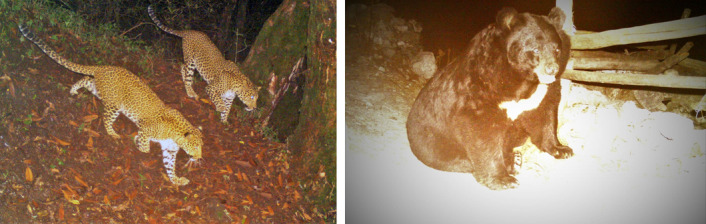

## 1 INTRODUCTION

Frequent interactions between people and wildlife often lead to negative behavior and conflicts (König et al., 2020). Human‐wildlife conflict (HWC) is a serious problem that affects both human and wildlife populations worldwide (Dowie, 2011), resulting in livestock predation, crop raiding, property damage, risks to human safety, and the retaliatory killing of wildlife (Nyhus, 2016; Ripple et al., 2015). Ultimately, when HWC are evaluated in terms of cost, human casualties emerge as the dominant factor (Gulati et al., 2021). For example, in the mid hills of Gandaki Province, Nepal, there were 77 reported cases of human causalities (69 human injuries and 8 deaths) from wildlife [common leopard (*Panthera pardus*) and Himalayan black bear (*Ursus thibetanus laniger*)] between 2011 and 2019 (Baral et al., 2021). The Government of Nepal compensates with Nepali Rupees (NRs) 1,000,000 for each human fatality, covering total injury costs as well (US$1 = NRs 130) (Annex S1). Even if the conflict species is not a large predator like a leopard, there can still be cultural disagreements regarding the risks of protecting these species (Redpath et al., 2015).

Nepal possesses one of the world's richest collections of biodiversity but faces constant pressure from agricultural development and over‐exploitation of wildlife (Monastersky, 2014). Cases of HWC in Nepal have been increasing, and Nepal is now recognized as one of the top 10 nations globally, with a significant occurrence of recorded HWC, accounting for approximately 10% of the total cases worldwide (Torres et al., 2018). For example, in Nepal, there were 3647 reported cases of HWC from 2007 to 2016 AD, resulting in 190 fatalities and the destruction of crops worth over NRs 1.2 billion (approximately US$11 million; US$1 = NRs 130). The wild boar (*Sus scrofa*), nilgai (*Boselaphus tragocamelus*), wild elephants (*Elephas maximus*), and rhino (*Rhinocerous unicornis*) were the four most frequent species cited in these instances (Timsina et al., 2012).

Nepal has adopted a range of strategies to address HWC, including the establishment of protected areas (PAs), the development of community‐based conservation programs, and the implementation of laws and policies to safeguard wildlife (Gurung et al., 2015, 2016; Timsina et al., 2012; Timsina & Goka, 2014). The HWC is not limited to protected areas but is widely distributed nationwide. For instance, two‐thirds of HWC instances in the past 5 years occurred outside of the PAs (Adhikari et al., 2018; Niraula et al., 2013). To address these conflicts, compensation is one of the most prevalent post‐conflict mitigation strategies adopted by the Government of Nepal (GoN). This approach involves providing monetary payments or non‐monetary rewards (Karki et al., 2016; Timsina et al., 2012). In 2022, NRs 20,62,000 (1US$ = NRs 130) were provided as compensation to families impacted by HWC within the Gaurishankar Conservation Area (GCA, 2022).

PAs were established to conserve key species like tigers (*Panthera tigris tigris*), Asian elephants, and rhinos in Nepal. They boast a substantial management budget and attract global researchers due to their charismatic wildlife, resulting in robust documentation of Human‐Wildlife Conflict within PAs (Acharya et al., 2016). Studies assessing aspects such as crop damage, livestock depredation, human casualties, and societal perceptions of wildlife have been conducted extensively within PAs (Bhatta & Joshi, 2021; Dhungana et al., 2016; Pant et al., 2023; Sapkota et al., 2014; Subedi et al., 2020). In contrast, HWC studies, particularly in conservation areas and high‐altitude regions, are limited due to budget constraints and the challenging nature of research in remote environments (Baral et al., 2021; Bista & Song, 2022; CBS, 2021; Sharma et al., 2021). In these high‐altitude regions, characterized by rich biodiversity and heavy reliance on natural resources for livelihoods, HWC commonly manifests as crop raiding, property damage, livestock depredation, and human injuries/deaths, with a focus on common leopards and Himalayan black bears (Adhikari et al., 2018; Baral et al., 2021; Bista & Aryal, 2013). This study contributes to understand HWC dynamics, emphasizing livestock depredation and human injuries/deaths in the Nepal Himalayas' high‐altitude regions.

A comprehensive grasp of HWC proves vital for conservation managers, especially in regions with constrained conservation budgets like Nepal and comparable developing countries (Dhakal et al., 2020). Understanding HWC patterns and drivers and identifying key conflict species and areas, particularly in less‐explored regions like the Gaurishankar Conservation Area (GCA), is crucial (Awasthi & Singh, 2015; Karanth et al., 2004). Analyzing HWC compensation data from GCA can provide insights into the current HWC situation and guide the formulation of effective mitigation measures. Notably, the amendment to the relief policy between BS 2073 and 2076 (2015–2018 AD; BS stands for Bikram Sambat, for which a new year day is in mid‐April AD) streamlined and enhanced support mechanisms for affected individuals, potentially influencing the reporting, and claiming of compensation for HWC incidents (Annexes S1 and S2). This is particularly relevant to high‐altitude conservation areas, which play a pivotal role in conservation and development efforts, given their natural habitats and importance in supporting rural livelihoods.

## 2 MATERIALS AND METHODS

### 2.1 Study area

**FIGURE 1:**
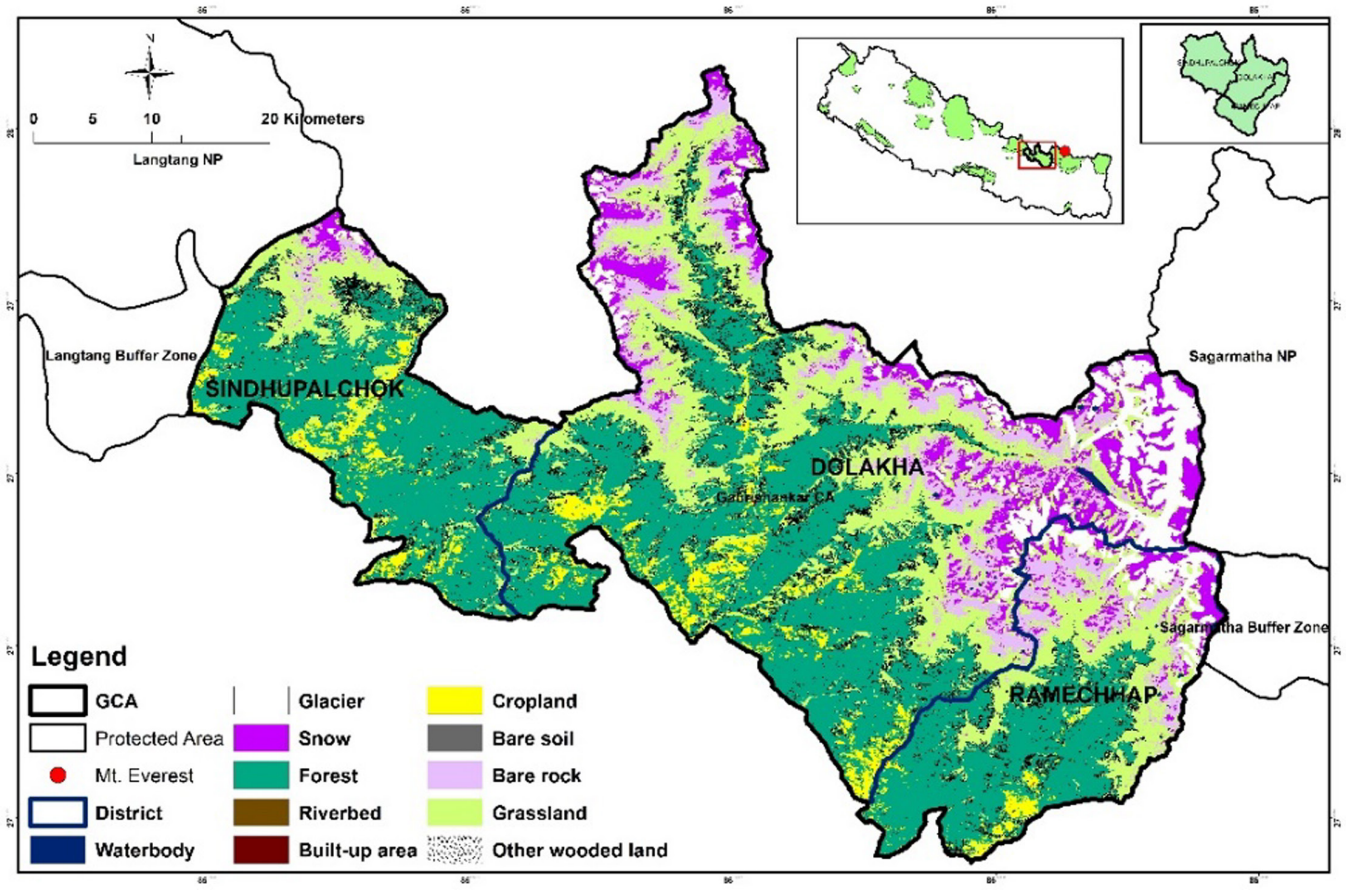
Map of the study area: Gaurishankar Conservation Area. The districts are shown on the top right side.

Our study area is the Gaurishankar Conservation Area (GCA), which was established in BS 2066 (2010 AD) (Figure 1) is a high‐elevation conservation area (range: 968 m–7181 m a.m.s.l.) in the northern part of central Nepal. “Conservation Area” means an area to be managed according to an integrated plan for the conservation of the natural environment and balanced utilization of natural resources (Act, 1973). It differs from national parks, as human activities are more restricted in national parks. The GCA has an area of 2179 sq km and encompasses two municipalities and eight rural municipalities of three districts: Dolakha, Sindhupalchok, and Ramechhap, and a snow‐capped peak of the Gaurishankar Mountain (7181 m). It is bordered by two important protected areas of the country, Sagarmatha National Park (which includes the world's highest peak: Mount Everest) in the east, Langtang National Park in the west, and China in the north (DNPWC, 2023). It is a renowned tourist destination and transit point of the trekking route that links Jiri Municipality, a popular tourist destination within the Dolakha district, to Sagarmatha (Mount Everest) National Park. Currently, the GCA is managed by the Gaurishankar Conservation Area Project (GCAP) under the National Trust for Nature Conservation, Nepal for 20 years (NTNC, 2023).

GCA encompasses diverse vegetation types: forests and bushes 44.5%, cultivated land 8.8%, grasslands 8.6%, glaciers 2.77%, and the rest are barren land, cropland, built‐up area, and others (Figure 1). The conservation area supports a remarkable diversity of 565 plant species, 78 mammal species, 16 fish species, 9 amphibian species, 22 reptile species, and 235 bird species (Chetri et al., 2022; NTNC, 2023; Pathak et al., 2022). Despite this, the abundance of large‐sized prey species (e.g., blue sheep *Pseudois nayaur*) is low in the region (Chetri et al., 2017).

The human population density of Dolakha, Sindhupalchok, and Ramechhap districts was 79, 103, and 110 persons/km^2^ in 2021. Emigration occurred across all districts, with average growth rates of −0.74%, −0.88%, and −1.67% respectively during 2021. The per capita income of Dolakha, Sindhupalchok, and Ramechhap districts were $922, $1110, and $951, respectively, while the per capita national income was $1196 in 2021. Agriculture, forestry, and fisheries were the most common occupations (~71–72%) within each of the three districts (CBS, 2021).

Guarding practices for livestock in Nepali high‐altitude conservation areas are almost the same as traditional methods (Jackson et al., 1996). In high‐altitude conservation areas like GCA, it varies by animal type and season. In winter, sheep and goats graze mostly unattended, while in summer, shepherds oversee them with guard dogs at night. Lambs and kids remain near the shed (goth in Nepali) during the day and are corralled with their mothers at night. Yaks roam freely, and cattle forage nearby, returning to their shed in the evening. During winter, livestock stay in temporary forest shelters, while in spring, they graze in high‐elevation pastures away from crops. Protection increases during snowfall and birthing when animals are stall‐fed and closely monitored (Jackson et al., 1996). Additionally, practices like fencing, making loud noises or fires to chase away leopards or Himalayan black bears, and using scarecrows for bears are employed (Awasthi & Singh, 2015).

### 2.2 Human–wildlife conflict data

When a HWC incident is reported, a team comprising GCAP's technical staff, representatives from the conservation forest management sub‐committee and local government under the supervision of the project chief investigate the incident spot to identify the species involved, including details about the number of domesticated animals killed, humans injured/death, the date and location of the incident (by year and district name) as soon as possible. The team classified the conflict‐causing species by interviewing the local people and examining their pugmarks, which vary by species (Kolipaka, 2014). Additionally, camera traps were sometimes set up at kill sites to confirm the predator's identity, as they often return to their kills (Zafar‐ul Islam et al., 2014). For human cases, pugmarks are examined too, and incidents were self‐reported, as no humans were killed. The team prepares and submits the HWC incident report including required documents (Annex S2) to the project chief, who then forwards it to the recommendation committee for compensation. Based on the recommendation from the committee, the Gaurishankar Conservation Area Liaison Office under the Department of National Parks and Wildlife Conservation provides compensation to the victim or their beneficiary.

**FIGURE 2:**
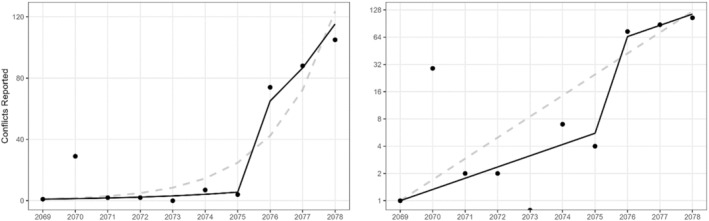
Total attacks reported per year by bear and leopard in Gaurishankar Conservation Area between BS 2069 and 2078 (2012–2021 AD) on the original scale (left) and log scale (right). On a log scale, the linear pattern of points suggests the number of reports per year is increasing exponentially. The black line shows the mean for the stochastic growth model increasing by a constant multiplicative factor (1.33), with a boost in reporting in BS 2076 (2019 AD) due to the policy change. The dashed gray line is a generalized linear model fit without a reporting boost in BS 2076 (2019 AD).

We used GCA records of incidents and compensation covering a period of 10 years [(BS 2069 to 2078) (2012–2021 AD)] to identify the species interacting with humans, the district with the most frequent HWC incidents, and the overall compensation provided. Initial exploratory analysis indicated that there were more incidents recorded after BS 2075 (2018 AD), which is referred to as the compensation policy change in our analysis.

### 2.3 Statistical analysis

We sought to measure the underlying rate of growth in reported conflicts over the period of monitoring, after accounting for any increase in reporting following the compensation policy change, as well as investigating how the number of incidents may differ by reporting region. Due to the limited amount of data (10 years), we restricted our analysis to estimating a single rate of change across the decade, with a single boost in reporting in BS 2076 (2019 AD), rather than estimating growth rates that vary before and after the rule change. We built a stochastic growth model to approximate the increase in conflict relative to the previous year (Figure 2), with parameters fit using maximum likelihood in R (version 4.3.0). The distribution of conflict $y_{i}$ during the $i^{\mathrm{th}}$ year of observation was modeled as Poisson, with a mean $\lambda_{i}$ that increases by a constant growth factor, $c_{1}$. Additionally, during the year 2076 (2019 AD), a fixed factor $c_{2}$ was added to account for the increase in attacks reported due to changes in policy:

$$y_{i}∼\text{Poisson}\left(\lambda_{i}\right)$$

For all years i except the first (BS 2069 or 2012 AD) and the year of the rule change (BS 2076 or 2019 AD):

$$\lambda_{i}=\lambda_{i-1}\cdot c_{1}$$

During BS 2076 (2019 AD), an additional factor of $c_{2}$ is included:

$$\lambda_{i}=\lambda_{i-1}\cdot c_{1}+c_{2}$$

**FIGURE 3:**
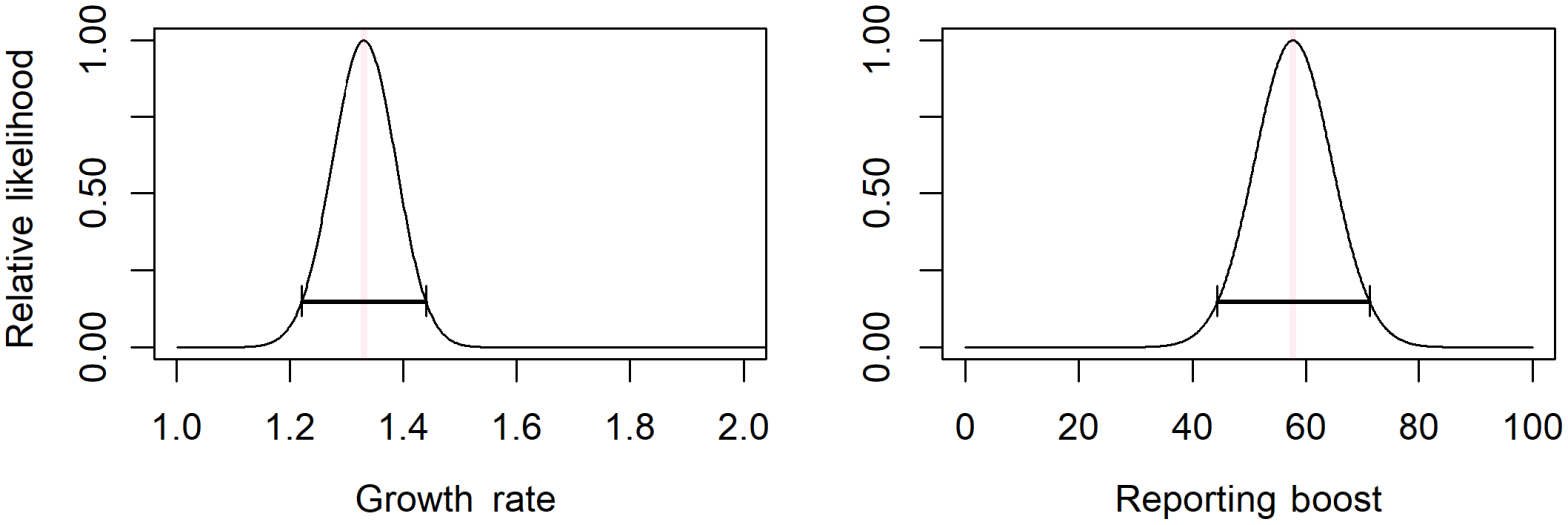
Relative profile likelihoods for parameters $c_{1}$ (left, growth rate) and $c_{2}$ (right) in the stochastic growth model with Poisson response. The maximum likelihood estimate is indicated by the vertical red bar, and the confidence interval (obtained by likelihood ratio test) indicated by the horizontal black bar.

Although this boost is applied in BS 2076 (2019 AD), the elevated reporting rate carries through to subsequent years since each year depends on the previous value. During the first year of record (BS 2069 or 2012 AD), the mean of the conflict was set equal to the initial observed conflict rate, or $\lambda_{1}=1.0$. Confidence intervals for parameters $c_{1}$ and $c_{2}$ were constructed using profile likelihood (Figure 3); however, these confidence intervals should be treated skeptically, as the number of observations was low, and confidence interval coverage becomes more accurate as the number of observations increased.

For comparison, we fit an equivalent generalized linear model (Poisson), but without a term to compensate for the rule change. In this case, we define the number of years after the start (BS 2069 or 2012 AD) as $x_{i}$, and use $x_{i}$ to control the mean:

$$y_{i}∼\text{Poisson}\left(\lambda_{i}\right)$$

$$\log\left(\lambda_{i}\right)=x_{i}\cdot c_{1}$$

We assess the quality of fit of the proposed stochastic growth model against the Poisson regression using a likelihood ratio test and the corrected Akaike information criterion (AICc), a small‐sample correction for the AIC (Bolker, 2008).

## 3 RESULTS

Between the years BS 2069‐078 (2012–2021 AD), there were 210 reported human‐wildlife conflicts (HWC) in the GCA. No human fatalities were reported. Out of the 210 cases, goats were the most targeted species, accounting for the majority (60% or 125 cases) of the incidents. Other livestock killed included cows/oxen (18%, 38 cases), yaks (9%, 19 cases), sheep (3%, 7 cases), and buffalo (~1%, 2 cases). Leopards were responsible for the specific killing of goats, whereas bears were only involved in direct attacks on humans (18 cases, or 90% of total human attack cases).

HWC reports were highest in the Dolakha district (88%, 186 cases), followed by Sindupalchowk (7%, 15 cases) and Ramechhap (4%, 9 cases) districts (Table 1). The total compensation amount for these years was NRs 2,43,6230. Livestock compensation made up most of this total (NRs 2,07,2000, 85%), while human injury compensation amounted to NRs 3,64,230 (15%). On average, compensation per case for human injury was around NRs 18,000, and for livestock was NRs 10,000. (US$1 = NRs130). Refer to data for details.

**TABLE 1:** District‐wise number of conflict reports for Himalayan black bear and common leopard in Gaurishankar Conservation Area, Nepal, between BS 2069 and 2078 (2012–2021 AD).

District	District area within GCA ($km^{2}$)	Bear	Leopard
Dolakha	1362.25	14	172
Ramechhap	410.09	4	5
Sindhupalchok	431.49	0	15

The number of attacks reported per year was estimated on average to increase by a factor of *c*_1_ = 1.33 each year, or a 33% increase over the previous year's value (95% confidence interval: 1.22–1.44) (Figure 3). Following the rule change in BS 2076 (2019 AD), we estimate that the number of attacks reported was *c*_2_ = 57, higher than expected due to a 33% increase over the BS 2075 (2018 AD) attack reports alone (95% confidence interval: 44–77) (Figure 3). The Poisson GLM estimated the number of reports to increase by a factor of 1.71 yearly (95% confidence interval: 1.68–1.76), substantially higher than the rate estimated when accounting for the change in compensation policy (Figure 2). Accounting for the compensation rule change within the model improved the AICc, reducing it from 227 to 178. The likelihood ratio test between the models also provided strong evidence that spending an additional degree of freedom on estimating the effect of the compensation change was worthwhile ($\chi_{1}^{2}=52.6,p=4.09E-13$).

**FIGURE 4:**
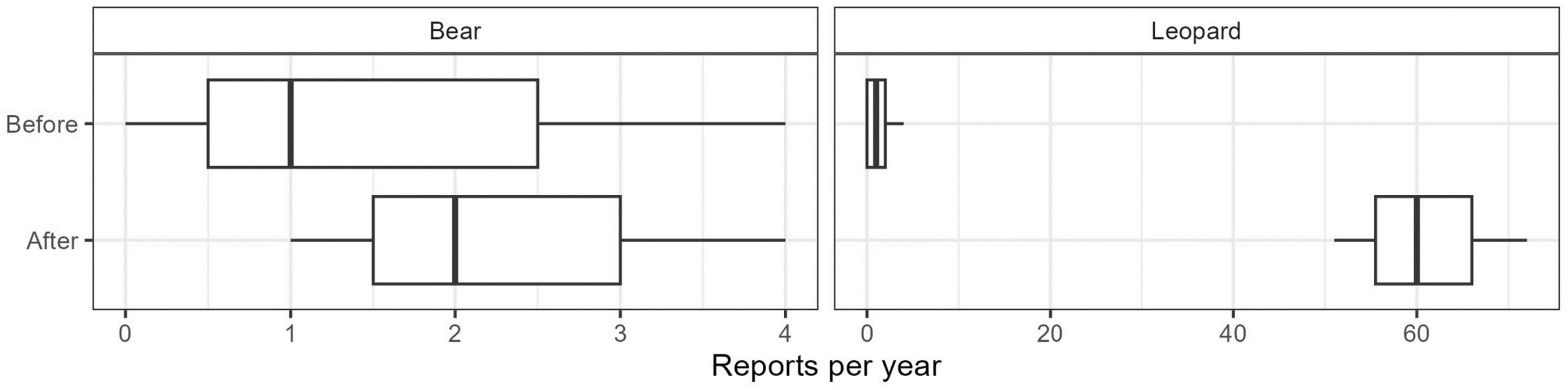
Boxplots of attacks reported per year within the GCA, by species, before and after the policy change.

The median number of bear attacks reported per year increased from one before the rule change to two afterward, but when compared using Welch's two‐sample *t*‐test, there was insufficient evidence to conclude that for mean reporting rate of bear attacks had been affected by the change of rules (*t* = 0.725, *p* = .5105) (Figure 4). On the other hand, the median number of leopard attacks reported per year increased dramatically from one to 60, with strong evidence that the reporting rate of leopard attacks has increased (*t* = 9.77, *p* = .0097) (Figure 4).

**FIGURE 5:**
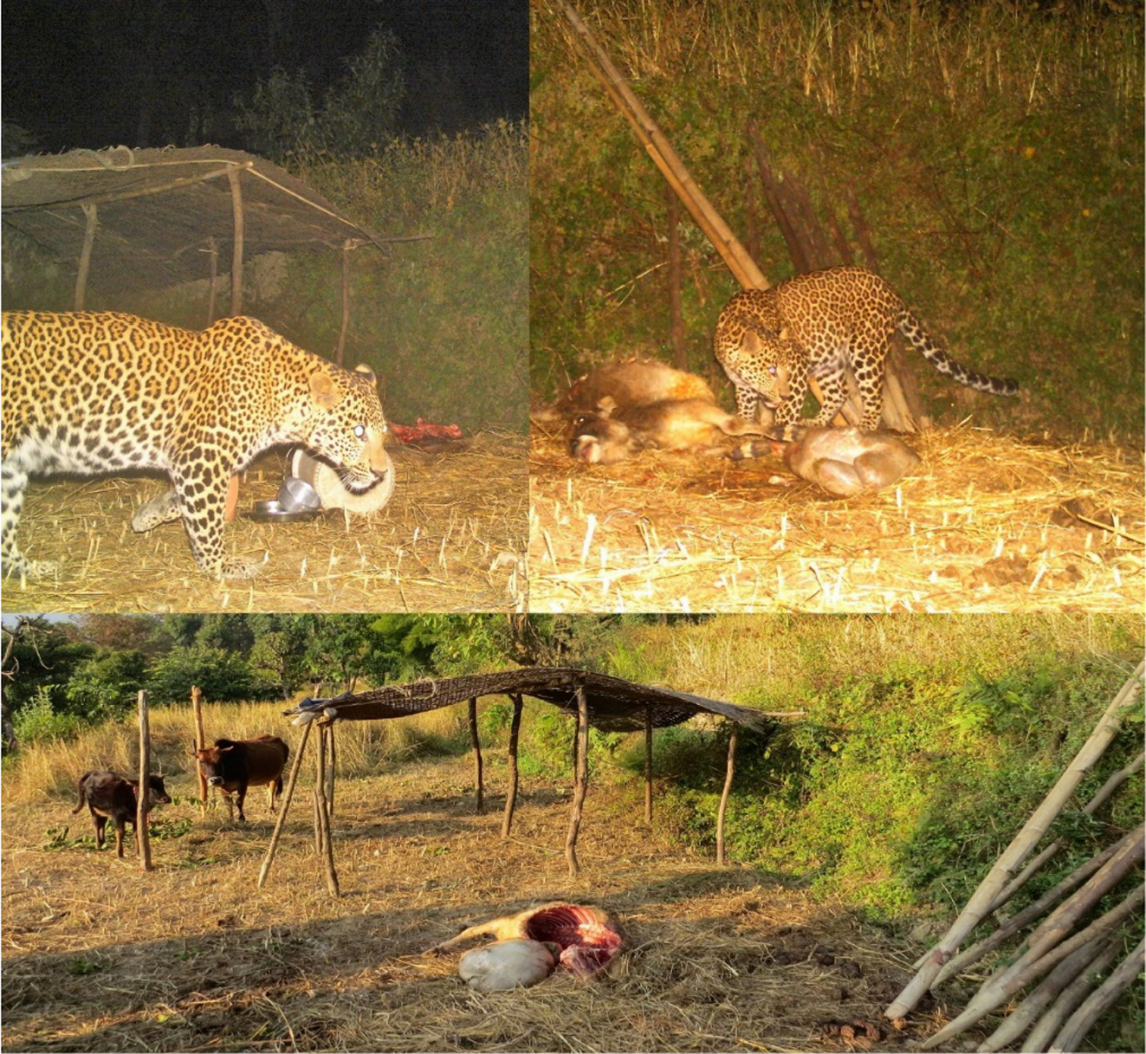
Photographs of livestock depredation by leopards in GCA (Source: DNPWC/GCAP/GCA, 2023). The largest single loss occurred when 24 goats were killed in a single incident in Dolakha during BS 2070 (2013 AD). For animals other than goats, the single largest loss was the killing of 10 yaks by a leopard in Sindhupalchok district during BS 2078 (2021 AD).

## 4 DISCUSSION

The study shed light on the prevalence and rate of increase in the reported human‐wildlife conflicts (HWC) within the GCA during the period between BS 2069 and 2078 (2012–2021 AD). The result showed a substantial increase in HWC incidents (33%), primarily driven by conflicts involving leopards and bears, with leopards being responsible for all livestock killings, while bears were involved in most cases of human injuries. Additionally, the compensation provided for the losses incurred emphasizes the economic impact of such conflicts on the local communities. Compensation for a single goat averaged NRs 6659 (US$51) up to as high as NRs 10,000 (US$76), however, an adult female goat may cost a farmer NRs 17,000 (US$130), and on around 25% of occasions, leopards would kill more than one goat (DOSL and JICA, 2017).

Leopards occupy a wide range of habitats in Nepal, spanning from lowland areas to mountain regions (Lamichhane et al., 2021; Stein et al., 2023). However, contrary to Baral et al. (2021), Lamichhane et al. (2018), and Acharya et al. (2016), there were only two recorded cases of human injuries and no human deaths by leopards. Since leopards are responsible for most livestock killings, it can be inferred that their presence is likely significant within the conservation area. While the exact occupancy and abundance of leopards in the Nepalese mountains remain uncertain, the adjacent Chure region in the lower south has an occupancy rate of 0.57 per 100 km^2^ (SE 0.0082) (Lamichhane et al., 2021).

Contrary to other predators that select prey based on their size (Andheria et al., 2007; Seidensticker & McDougal, 1993; Sunquist, 1981), leopards have a diverse diet that ranges from small rodents to prey like barking deer (*Muntiacus vaginalis*). Leopards may have targeted goats in this case due to their size, or local availability. Research has shown that predator dependency on livestock decreases with an increase in wild prey base (Biswas & Sankar, 2002; Reddy et al., 2004). The abundance of large‐bodied prey species (e.g., blue sheep) has previously been reported to be low in the region (Chetri et al., 2017), a factor that could potentially be improved by actions increasing the natural leopard prey population base.

The rise in goat depredation may in part due to the success of government agricultural credit and investment programs. The Government Small Farmer Development Program distributed NRs 12.88 billion (US$109 million) in loans during 2021, of which 36.7% were used for livestock and farming services. Approximately 65% of animals purchased during 2021 were goats (603,485 goats out of 925,758 total). Between 2015 and 2021, the purchase of 3 million goats was funded, a significant contribution to the goat population of Nepal, which in 2019 was 12.8 million (MoF, 2021).

Delving deeper, leopards entering loosely constructed barns have resulted in livestock killing (Figure 5). Constructing secure barns (corrals) can significantly minimize livestock attacks, however, the cost of shed construction is substantial, representing 40%–60% of the fixed costs of starting a goat farm (DOSL and JICA, 2017). This is especially difficult for the first year of operation, during which no goats or goat products are sold, and to compensate it is sometimes recommended that farmers improvise in building materials with scrap on hand. The walls, together with the beams, account for approximately 50% of construction costs, so it is not surprising that cost‐cutting in external protections would lead to insecure goat sheds. Our recommendation is to promote programs supporting predator‐proof corrals targeting economically marginalized groups, particularly in areas with sizable leopard populations (Jackson et al., 1996; Jackson & Wangchuk, 2004). Predator‐proof corrals are more costly to construct, but effectively protect livestock and boost the economic well‐being of people in the areas (Joshi, 2019). In addition, community education and awareness programs on wildlife conservation are crucial mitigation measures (Mkonyi, 2022).

Conflicts between humans and the Himalayan black bear are frequently observed in Nepali mid‐mountains, making it a significant issue in the region (Bista & Aryal, 2013). Our results confirm attacks by bears continue to pose a risk of minor and serious injury to humans in the GCA, with most bear attacks reported between October and December. Himalayan black bears usually hibernate between December to March and in preparation will eat more grains and fruit, venturing farther in search of food (Bashir et al., 2018), potentially leading to more frequent encounters and conflicts with humans. Hence, strategies for conflict mitigation such as the use of deterrents or secure enclosures, raising awareness among local communities, appropriate waste management, responsible food storage, and insurance programs could be suitable solutions (Joshi, 2019; Pathak et al., 2023; Somu & Palanisamy, 2022).

Acknowledging the perception and attitude of the local stakeholders toward wildlife is crucial for successful conservation programs (Pathak et al., 2023). To reduce negative public perceptions of wildlife, GoN formulated the Wildlife Damage Relief Support Program in 1998 (Annex S1). Although the waiting time for the relief amount is long in Nepal, the GCA has tried to promptly address compensation. For instance, in Chitwan National Park of Nepal, the wildlife victims had to wait for an average of 6.6 months to get relief (Lamichhane et al., 2019) compared to GCA, with less than a month waiting time (GCA, 2022). This accelerated relief distribution within the GCA has played a key role in motivating local communities toward active participation in wildlife conservation efforts. More broadly, the GoN has continually improved the regulatory directives, the latest being in 2023, which aims to further simplify the relief distribution procedure (Annexes S1 and S2), potentially making lessons learned about elevated reports and compensation changes within the GCA relevant to other conservation areas across Nepal in the near future.

## 5 CONCLUSION

Human–wildlife conflicts are a central focus in wildlife conservation, necessitating continuous attention and adaptation. Research plays a pivotal role in comprehending the complexities of HWC and in promoting and implementing diverse solutions for sustainable coexistence. Our study highlights the crucial role of an effective relief policy in accurately assessing the number of conflict cases. Understanding the actual occurrences enables the formulation of strategies aimed at enhancing human‐wildlife coexistence. The dynamics of conflicts may evolve, especially with changes in the human‐modified landscape that impact wildlife communities. Therefore, a timely revision of relief policies is imperative to address such shifts. Acknowledging the absence of a one‐size‐fits‐all solution, our proposed strategies include market‐based compensation, secured enclosures (corrals) for livestock, bolstering prey populations, community education, awareness initiatives, community‐based insurance programs, and collaborative efforts for alternative livelihood options. These multifaceted approaches collectively contribute to strengthening the coexistence between humans and wildlife, acknowledging the ever‐changing nature of HWC challenges.

## AUTHOR CONTRIBUTIONS

**Abhinaya Pathak:** Conceptualization (equal); investigation (equal); methodology (equal); resources (equal); supervision (equal); validation (equal); visualization (equal); writing – original draft (equal); writing – review and editing (equal). **Saneer Lamichhane:** Conceptualization (equal); formal analysis (equal); methodology (equal); software (equal); writing – original draft (equal); writing – review and editing (equal). **Maheshwar Dhakal:** Resources (equal); supervision (equal); writing – review and editing (equal). **Ajay Karki:** Resources (equal); supervision (equal); writing – review and editing (equal). **Bed Kumar Dhakal:** Resources (equal); supervision (equal); writing – review and editing (equal). **Madhu Chetri:** Resources (equal); supervision (equal); writing – review and editing (equal). **Jeffrey Mintz:** Software (equal); validation (equal); visualization (equal); writing – review and editing (equal). **Prakash Pun:** Conceptualization (equal); investigation (equal); project administration (equal); writing – original draft (equal); writing – review and editing (equal). **Pramila Neupane:** Conceptualization (equal); data curation (equal); validation (equal); writing – review and editing (equal). **Tulasi Prasad Dahal:** Resources (equal); supervision (equal); validation (equal); writing – review and editing (equal). **Trishna Rayamajhi:** Data curation (equal); methodology (equal); writing – review and editing (equal). **Prashamsa Paudel:** Supervision (equal); validation (equal); writing – review and editing (equal). **Pramod Raj Regmi:** Data curation (equal); supervision (equal); validation (equal); writing – review and editing (equal). **Shankar Thami:** Data curation (equal); supervision (equal); validation (equal); writing – review and editing (equal). **Ganesh Thapa:** Data curation (equal); resources (equal); validation (equal); writing – review and editing (equal). **Ashim Thapa:** Writing – review and editing (equal). **Suraj Khanal:** Data curation (equal); resources (equal); supervision (equal); validation (equal); writing – review and editing (equal). **Supriya Lama:** Data curation (equal); resources (equal); supervision (equal); validation (equal); writing – review and editing (equal). **Jenisha Karki:** Data curation (equal); resources (equal); supervision (equal); validation (equal); writing – review and editing (equal). **Sujan Khanal:** Visualization (equal); writing – review and editing (equal). **Arockia E J Ferdin:** Writing – review and editing (equal).

## FUNDING INFORMATION

None.

## CONFLICT OF INTEREST STATEMENT

There is no conflict of interest.

### OPEN RESEARCH BADGES

This article has earned Open Data, Open Materials and Preregistered Research Design badges. Data, materials and the preregistered design and analysis plan are available at [https://doi.org/10.5061/dryad.0zpc86750].

## Supporting information


Annexes S1‐S2

## Data Availability

All data of this research is accessible: https://datadryad.org/stash/share/bBc6PujPTt_lgTAF9xW38essT3WmU_2sI_j‐4XmYvrU
