# Correction to “Human‐wildlife conflict at high altitude: A case from Gaurishankar conservation area, Nepal”

**DOI:** 10.1002/ece3.70119

**Published:** 2024-08-07

Pathak, A., Lamichhane, S., Dhakal, M., Karki, A., Dhakal, B.K., Chetri, M., Mintz, J., Pun, P., Neupane, P., Dahal, T.P., Rayamajhi, T., Paudel, P., Thapa, A., Regmi, P.R., Thami, S., Thapa, G., Khanal, S., Lama, S., Karki, J., Khanal, S. and Ferdin, A.E.J. (2024). Human‐wildlife conflict at high altitude: A case from Gaurishankar Conservation Area, Nepal. *Ecology and Evolution*, *14*(7), p.e.11685, p1‐9. 10.1002/ece3.11685

Figure citations in the following paragraphs have been corrected in the article.

1. In Paragraph 1 of section 2.2 “Human‐Wildlife Conflict Data” Figure 2 mentioned in the text have been deleted.
2. In Paragraph 1 of section 2.3 “Statistical analysis,” Figure 2 has been added in the bracket after the “year” in the sentence “We built a stochastic growth model to approximate the increase in conflict relative to the previous year” **(Figure 2)**.
3. In Paragraph 3 of the Result section, Figure 3 has been added in the bracket after the sentences “The number of attacks reported per year was estimated on average to increase by a factor of c_1_ = 1.33 each year, or a 33% increase over the previous year's value (95% confidence interval: 1.22–1.44) (**Figure 3)**”; and “Following the rule change in BS 2076 (2019 AD), we estimate that the number of attacks reported was c_2_ = 57, higher than expected due to a 33% increase over the BS 2075 (2018 AD) attack reports alone (95% confidence interval: 44–77) **(Figure 3).**”
4. In Paragraph 3 of the Result section, Figure 4 has been corrected to Figure 2 in the sentence “The Poisson GLM estimated the number of reports to increase by a factor of 1.71 yearly (95% confidence interval: 1.68–1.76), substantially higher than the rate estimated when accounting for the change in compensation policy (**Figure 2**).”
5. In Paragraph 4 of the Result section, Figure 5 has been corrected to Figure 4 in sentence “….. been affected by the change of rules (*t* = 0.725, *p* = .5105) (**Figure 4**). On the other hand, the median number of leopard attacks reported per year increased dramatically from one to 60, with strong evidence that the reporting rate of leopard attacks has increased (*t* = 9.77, *p* = .0097) (**Figure 4**).”
6. In Paragraph 5 of the Discussion section, Figure 2 has been corrected to Figure 5 in the sentence “Delving deeper, leopards entering loosely constructed barns have resulted in livestock killing (**Figure 5**).”

We apologize for these errors.

