# Supplementary material for: Human‐wildlife conflict at high altitude: A case from Gaurishankar conservation area, Nepal
Source: Ecol Evol. 2024 Jul 8;14(7):e11685. doi: 10.1002/ece3.11685 (PMC11367734; doi:10.1002/ece3.11685)
Supplement: Supplementary file 1 — Annexes S1‐S2 [file ECE3-14-e11685-s001.pdf]

Annex 1: Major Relief support provision in Nepal with amendment timeline

S.N.	Details	Chitwan National Park (Buffer zone Management committee-2054/12/18) for CNP only	Wildlife damage relief support directive, 2066	Wildlife damage relief support directive, 2069	Wildlife damage Relief support directive, 2069 (First Amendment, 2072)	Wildlife damage relief support directive, 2069 (Second Amendment, 2074)	Wildlife damage Relief support directive, 2069 (Third Amendment, 2075)	Wildlife Damage Relief distribution directive, 2080	Remarks
**1.**	**Human Casualty**								*If the expenditure exceeds the limit, additional expenditure will be managed by government of Nepal on the recommendation of particular hospital.<br>** If victim is seriously disable for life, then, NPR. 5,00,000 for the victim or the victim's family as for livelihood.
	a. Minor Injury	5000	5000	10000	10000	20000	20000	20000	
	b. Serious Injury	10000	50000	50000	100000	200000	200000*	200000* (**)	
	c. Death	25000	1,50,000	300000	500000	1000000	1000000	1000000	
**2.**	**Domesticated animal damage**								

	a. Adult Buffalo, He-Buffalo, Improved cow and Ox species	-	-	-	30000	30000	30000	60000	# Fish and Poultry damage relief added under domesticated animal.
	b. Other domesticated animals	25-50 % of their registered value	10000	10000	10000	10000	10000	10000 (#)	
**3.**	**House and Shed house**								
	a. House and shed house	25-50 % of their registered value	4000	10000	10000	10000	10000	20000	
	b. Stored Grains		5000	10000	10000	10000	10000	10000	
4.	**Crop damage**	0	5000	10000	10000	10000	10000	10000°	Crop also include Sugarcane and Professional banana cultivation (Since first amendment)<br>°All crops damage cultivated in private lands
5.	**Wild animal species that can produce**	n/a	n/a	**8 species**<br>1. Elephant,<br>2. Rhino,<br>3. Tiger,<br>4. Bear<br>5. Leopard,	**11 species**<br>1. Elephant,<br>2. Rhino,<br>3. Tiger,<br>4. Bear<br>5. Leopard,	**14 species** 1. Elephant,<br>2. Rhino,<br>3. Tiger,<br>4. Bear<br>5. Leopard,	**14 species**<br>1. Elephant,<br>2. Rhino,<br>3. Tiger,<br>4. Bear<br>5. Leopard,	**16 species**<br>1. Elephant,<br>2. Rhino,<br>3. Tiger,<br>4. Bear<br>5. Leopard,	

	**relief support**			6. Snow Leopard,<br>7. Clouded leopard<br>8. Wolf	6. Snow Leopard,<br>7. Clouded leopard<br>8. Wolf<br>9. ***Wild dog***<br>***10. Wild boar***<br>***11. Wild water buffalo***	6. Snow Leopard,<br>7. Clouded leopard<br>8. Wolf<br>9. Wild dog<br>10. Wild boar<br>11. Wild water buffalo<br>12. ***Mugger Crocodile***<br>***13. Gaur (Bison)***<br>***14. Python***	6. Snow Leopard,<br>7. Clouded leopard<br>8. Wolf<br>9. Wild dog<br>10. Wild boar<br>11. Wild water buffalo<br>12. Mugger Crocodile,<br>13. Gaur (Bison)<br>14. Python	6. Snow Leopard,<br>7. Clouded leopard<br>8. Wolf<br>9. Wild dog<br>10. Wild boar<br>11. Wild water buffalo<br>12. Mugger Crocodile,<br>13. Gaur (Bison)<br>14. Python<br>15. ***Blue Bull***<br>***16. Monkey***	
6.	**Relief providing agency**	Chitwan National Park (Buffer zone Management committee	Regional Forest Directorate	Regional Forest Directorate	Regional Forest Directorate	Regional Forest Directorate	Protected Area Office	Protected Area Office<br>& Divisional Forest Office	
7.	**Time to provide relief**	n/a	Not mentioned	Not mentioned	Not mentioned	Not mentioned	Not mentioned	Within 1 Month	
8.	**For non-relief producing wild animal species**	n/a	n/a	Based on the directive, Buffer zone management committee, conservation area management council and Community	Based on the directive, Buffer zone management committee, conservation area management council, Community	Based on the directive, Buffer zone management committee, conservation area management council, Community	Based on the directive, Buffer zone management committee, conservation area management council, Community Forest user	Buffer zone management committee, Conservation area management committee, conservation area management council,	

				Forest user group can provide relief amount.	Forest user group, DFO and organization registered in protected area can provide relief amount. Further, conservation partners can provide additional relief support in case of death and serious injury.	Forest user group, DFO and organization registered in protected area can provide relief amount. Further, conservation partners can provide additional relief support in case of death and serious injury.	group, DFO and organization registered in protected area can provide relief amount. Further, conservation partners can provide additional relief support in case of death and serious injury.	Community Forest user group, Buffer zone user committee, Conservation area management committee, Buffer zone community forest user group or other forest user group have to allocate at least 5% of their internal fund for wildlife damage relief purpose. This fund and other fund available can be used to provide relief for the damage caused by animals other than listed in S.N. (5) of this table.	

Note: Monetary values are in Nepali Currency and dates are in Nepali.

Annex 2: Existing process to distribute Wildlife damage relief support in Nepal

1. Victim must submit letter to protected area office or Division forest office for relief support with required documents within 35 days of incident

2. Based on the decision made by relief support recomendation committee, relief is provided to the vicitm by Protected area or Division forest office, if possible through banking system.

3. Relief support can be provided to their close one/dependents (in case of death) while by themselves or agreed letter by victim to provide to their preferred individuals.

Required documents:

- Relief support letter
- Photo of victim or dead individual (if possible)
- Death Certificate
- Incident minute/report
- Post Mortem Report
- Police report
- Community user group or committee recommendation letter
- Relation proof document
- Health institution receipt and recommendation letter
- Evaluation by Veterinary or agriculture service office
- Land ownership proof

Timeline: Victim should receive the relief amount within 1 month

Recommendation committee:

- Protected area chief or Divisional Forest officer
- Community user committee chair
- Community forest user committee chair
- Local government representative or chief administrative officer
- Representative from Veterinary or agriculture service office (according to the damage)
- If necessary, experts can be invited
